# Bilateral multiple stroke, left upper extremity ischemia, and transient complete atrioventricular block in transcatheter aortic valve implantation: a case report

**DOI:** 10.1186/s40981-023-00669-x

**Published:** 2023-11-07

**Authors:** Yuki Mitsuta, Shingo Nakamura, Yumiko Uemura, Koichiro Tashima, Takafumi Oyoshi, Naoyuki Hirata

**Affiliations:** 1 Department of Anesthesiology, Japanese Red Cross Kumamoto Hospital, Kumamoto, Japan; 2 Department of Anesthesiology, Kumamoto University Hospital, 1-1-1, Honjo, Chuo-Ku, Kumamoto, 860-8556 Japan; 3 Department of Anesthesiology, Minamata City General Hospital & Medical Center, Minamata, Japan

**Keywords:** Transcatheter aortic valve implantation, Stroke, Embolism

## Abstract

**Background:**

Transcatheter aortic valve implantation (TAVI) is a minimally invasive surgery. However, there is a risk of surgical manipulation causing detachment of a lesion of the aortic valve, which can result in various embolisms.

**Case presentation:**

An 87-year-old woman with symptomatic severe aortic valve stenosis was scheduled for transfemoral TAVI under monitored anesthesia. Preoperative examination revealed severe calcification of the aortic valve, but there was no calcification in the ascending aorta. After a delivery catheter system passed the aortic valve, left radial arterial pressure dropped significantly, and complete atrioventricular block (CAVB) occurred. Catecholamine administration and ventricular pacing improved hemodynamics, and a self-expandable valve was implanted. CAVB resolved after surgery, but her state of consciousness was poor, and her left hand became ischemic. Imaging studies revealed multiple embolic infarcts in her bilateral cerebrum and cerebellum.

**Conclusions:**

It should be noted that there is a risk of detachment of a calcified lesion of the aortic valve during TAVI, which can cause embolisms not only in the brain but also in the extremities and coronary arteries.

## Background

Transcatheter aortic valve implantation (TAVI) is a minimally invasive surgery for patients with aortic valve stenosis (AS). However, one of the serious complications is stroke. The frequency of stroke after TAVI is 0.6–5.5% [1–6], and most of the cases of stroke are due to embolism [7]. Patients undergoing TAVI often have calcification in the aortic valve and aorta. Surgical manipulation can cause detachment of calcified debris, which can result in cerebral embolism. Calcified debris can also cause embolism in organs other than the brain, but there are few such case reports.

In this report, we describe a case of postoperative bilateral stroke, left upper extremity ischemia, and intraoperative complete atrioventricular block (CAVB), which might have been embolisms caused by the release of a calcified lesion of the aortic valve by surgical manipulation in TAVI.

## Case presentation

**Fig. 1:**
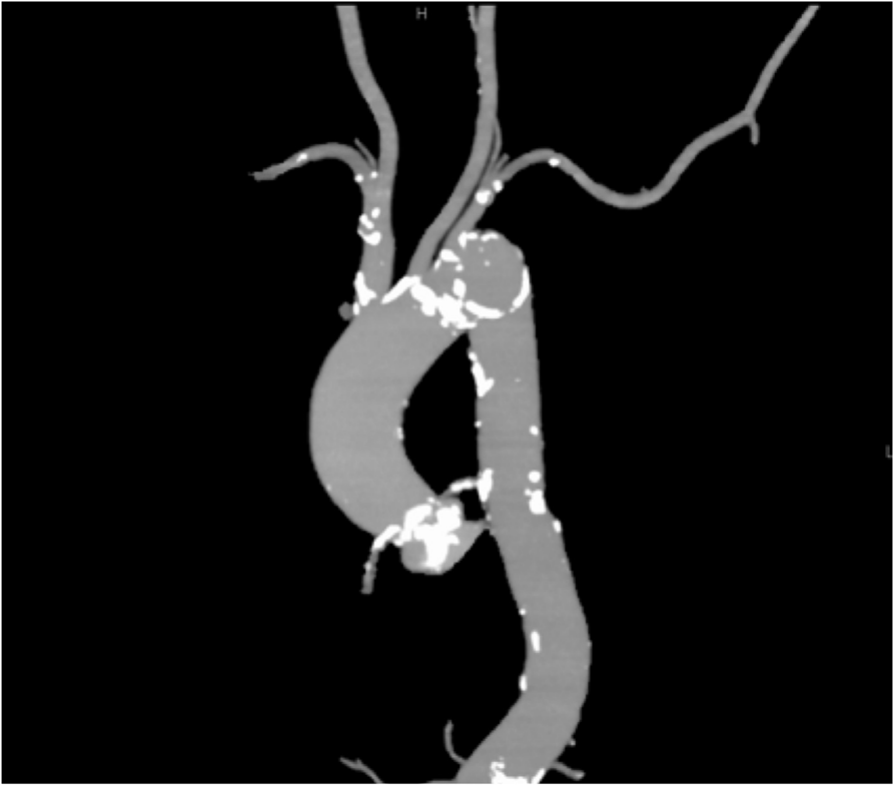
Preoperative computed tomography shows severe calcification of the aortic valve and scattered calcification in the aorta

An 87-year-old woman was admitted to our hospital with heart failure and was diagnosed with severe aortic valve stenosis (AS) shown by transthoracic echocardiography (TTE). While her medical history included cerebellar infarction 4 years ago and left anterior descending artery stenosis with percutaneous coronary intervention performed 1 month ago, she had no clinical symptoms on admission. An electrocardiogram (ECG) showed sinus rhythm and no conduction disturbance. TTE showed an ejection fraction of 72%, good left ventricular wall motion, mild left ventricular hypertrophy, very severe AS (aortic valve area: 0.49 cm^2^, maximum velocity: 5.5 m/s, and mean pressure gradient: 81 mmHg), and trivial aortic valve regurgitation (AR). Computed tomography (CT) revealed a highly calcified aortic valve and scattered calcification in the aortic arch, descending aorta, and abdominal aorta (Fig. 1). We planned transfemoral TAVI with a self-expandable valve (Evolut PRO +) under monitored anesthesia.

**Fig. 2:**
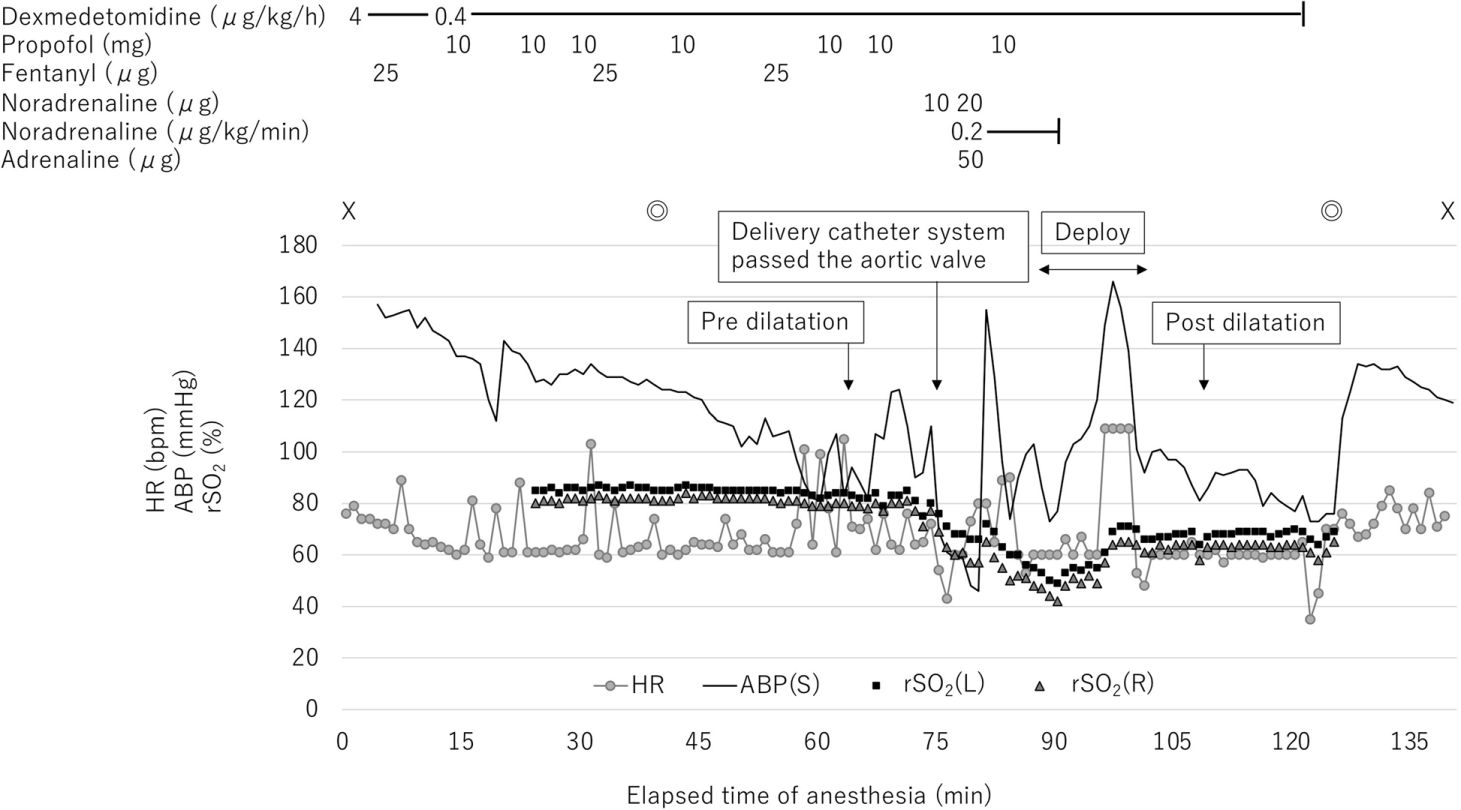
Anesthesia record. X, start and end of anesthesia; ◎, start and end of the operation; HR, heart rate; ABP(S), systolic arterial blood pressure; rSO_2_(L), left regional oxygen saturation; rSO_2_(R), right regional oxygen saturation

**Fig. 3:**
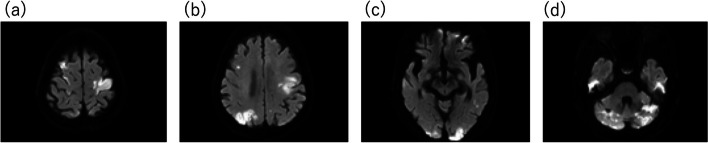
Diffusion-weighted magnetic resonance images 5 h after surgery. Multiple embolic infarcts in the bilateral frontal (**a**), parietal and occipital lobes (**b** and **c**), and the cerebellar hemispheres (**d**) were detected

Administration of dexmedetomidine was maintained at 0.4 μg/kg/h, and propofol and fentanyl were administered as needed (Fig. 2). An arterial blood pressure (ABP) line was inserted into the left radial artery, and a temporary pacemaker was placed through the right internal jugular vein. We used bispectral index (BIS) for evaluation of the level of anesthesia depth and monitored cerebral regional oxygen saturation (rSO_2_) for evaluation of cerebral blood flow and oxygenation. Heparin was administered, and activated clotting time was maintained at 250 s or more. Pre-dilatation was performed under rapid pacing at 180 bpm. There were no ECG changes, pericardial effusion, or exacerbation of AR. As the delivery catheter system passed through the aortic valve and the position was being adjusted, complete atrioventricular block (CAVB) occurred, and ABP dropped significantly. The pulse wave of the pulse oximeter attached to the left hand became undetectable. However, femoral artery pulsations were palpable. Immediately, ventricular pacing was started; noradrenaline was administered as a bolus followed by a continuous infusion. Additionally, 50-μg adrenaline was administered. During CAVB, left ventricular wall motion was normal in TTE, but right ventricular wall motion could not be examined. Abnormal coronary artery flow during CAVB was also not detected by intraoperative coronary angiography. ABP was improved, but BIS and bilateral rSO_2_ decreased (left: 85 to 50, right: 80 to 42). A self-expandable valve was implanted, and the surgery was completed. CAVB reverted to normal sinus rhythm. However, recovery of consciousness was delayed, and her extremity movements were inadequate. Furthermore, her left hand was pale and felt cold. Magnetic resonance imaging (MRI) revealed multiple embolic infarcts in the bilateral frontal, parietal and occipital lobes, and cerebellar hemispheres (Fig. 3). However, angiography showed no treatable vessel occlusion.

On postoperative day 1, ST-T depression in II, III, and aVF was observed, and high-sensitivity troponin T (hsTnT) was increased compared to preoperative value (hsTnT: 0.0212 to 0.348 μg/mL).

The left-hand ischemia gradually improved, but the right upper extremity paralysis due to stroke persisted.

## Discussion

During TAVI, as the delivery catheter system passed through the aortic valve and the position was being adjusted, CAVB occurred, left radial ABP dropped, the waveform of the pulse oximeter attached to the left hand disappeared, and BIS and bilateral cerebral rSO_2_ decreased. After TAVI, left upper extremity ischemia and bilateral multiple cerebral embolisms were revealed. We considered that these events may have occurred through a unitary mechanism.

TAVI has been widely performed and is becoming safer, but the incidence of postoperative stroke has not decreased. This may be related to characteristics specific to patients with severe AS. It has been reported that patients who experience TAVI-related stroke tend to be female, have a history of stroke, and have peripheral vascular disease, ischemic heart disease, or renal failure [8]. Our case also had many of these characteristics.

TAVI involves manipulation of the calcified aortic valve and aortic wall by using a delivery catheter system, pre-dilatation, positioning and implantation of the prosthetic valve, and post-dilatation. Therefore, detachment of fragments of the calcified native valve and the aorta may occur, leading to cerebral embolism [9]. One study with transcranial Doppler in TAVI showed that cerebral embolism often occurs during prosthetic valve positioning and valve deployment [10]. Aortic valve calcium score, measured by CT, has been reported to be an independent risk factor for TAVI-related stroke [11]. Our case had a high aortic valve calcium score of 3140 Agatston units, suggesting that the patient was at a high risk of stroke. Calcification of the left ventricular outflow tract and calcification of the aortic arch have also been reported as predictive factors for TAVI-related stroke [12, 13]. Conversely, it has also been reported that calcification of the aortic valve predicted an increase of white matter hyperintensity volume, while calcification of the aortic arch and calcification of the left ventricular outflow tract are not associated with an increase in white matter hyperintensity volume after TAVI [14].

In our case, calcified lesions were observed in the aortic valve, aortic arch, and descending aorta, but cerebral emboli occurred bilaterally in the cerebrum and cerebellum. Therefore, we believe that the source of the embolism might have been located more proximally than the first branch of the aortic arch, that is, the emboli were calcified lesions of the aortic valve.

In TAVI, a calcified aortic valve can embolize organs other than the brain. Considering that calcified lesions disperse to the arch branch, it is quite conceivable that emboli disperse from the subclavian artery to the upper extremity arteries and cause embolism. However, there has been only one case report of a calcified lesion of the aortic valve causing limb arterial embolism [15]. In our case, left radial arterial pressure decreased, and the pulse wave of the pulse oximeter attached to the left hand weakened during surgery, and left-hand ischemia was revealed postoperatively. It is thought that the detachment of the aortic valve lesion caused left upper extremity embolism in the same way as the cerebral embolism. Since the attenuation of the radial arterial pressure improved during the operation, upper extremity angiography was not performed. The left-hand ischemia was fortunately resolved without intervention, but imaging studies would be necessary for a definitive diagnosis and treatment.

CAVB can occur in TAVI mainly because the prosthetic valve physically impairs the conduction system, but transient CAVB can also occur when the left ventricular wire crosses the aortic valve [16]. The incidence of postoperative pacemaker implantation is as high as 3.8–25.9% [1–6], and there are various predictive factors for pacemaker implantation including preoperative conduction disturbances, use of self-expandable valves, and depth of valve placement [17]. However, in this case, the timing of CAVB was not after deployment of the prosthetic valve but during valve position adjustment. Considering that the calcified lesion of the aortic valve caused cerebral and upper extremity embolisms, CAVB may also have been an embolism caused by dispersal of calcified debris, with embolism to the atrioventricular branch of the right coronary artery. While abnormal findings were not detected in ST-T of ECG, TTE, and coronary angiography during the events, ST-T depression in II, III, and aVF was observed on the postoperative day 1. In addition, high-sensitive troponin T was increased on postoperative day 1 compared to preoperative value (hsTnT: 0.0212 to 0.348 μg/mL). Those results suggest that right coronary artery ischemia, which might have been caused by dispersal of calcified debris from valve lesions, occurred intraoperatively.

As far as we have investigated, we have not found any reports of CAVB due to embolism from valve lesions, but it is important to assume that possibility.

## Conclusions

We reported a case of postoperative multiple stroke, left upper extremity ischemia, and intraoperative CAVB in TAVI. Careful attention must be paid to the risk of various embolisms caused by a calcified lesion of the aortic valve.

## Data Availability

Data sharing is not applicable to this article as no datasets were generated or analyzed during the current study.

## Abbreviations

TAVI: Transcatheter aortic valve implantation
AS: Aortic valve stenosis
CAVB: Complete atrioventricular block
TTE: Transthoracic echocardiography
ECG: Electrocardiogram
AR: Aortic valve regurgitation
CT: Computed tomography
ABP: Arterial blood pressure
BIS: Bispectral index
rSO_2_: Regional oxygen saturation
MRI: Magnetic resonance imaging
